# Binge eating behavior in a sample of Lebanese Adolescents: Correlates and Binge Eating Scale validation

**DOI:** 10.1186/s40337-021-00493-7

**Published:** 2021-10-20

**Authors:** Anthony Mina, Souheil Hallit, Radoslaw Rogoza, Sahar Obeid, Michel Soufia

**Affiliations:** 1 Faculty of Medicine and Medical Sciences, Holy Spirit University of Kaslik (USEK), Jounieh, Lebanon; 2 Research Department, Psychiatric Hospital of the Cross, Jal Eddib, Lebanon; 3 Institute of Psychology, Cardinal Stefan Wyszyński University, Wóycickiego 1/3, 01-938 Warsaw, Poland; 4 Faculty of Arts and Sciences, Holy Spirit University of Kaslik (USEK), Jounieh, Lebanon

**Keywords:** Binge Eating, Adolescents, Body dissatisfaction, Self-esteem, Depression

## Abstract

**Background:**

Binge eating disorder is a common eating disorder among the adolescent population. The available literature in the Middle East in general, and Lebanon specifically, is relatively scarce and/or outdated. The objectives of this study were to (1) validate the Binge Eating Scale (BES) for use in Lebanese adolescents, and (2) assess correlates of binge eating behavior among this population.

**Methods:**

A cross-sectional study conducted between May and June 2020, enrolling 555 adolescents between the ages of 15–18 years old from all Lebanese governorates. The Binge Eating Scale was used to screen for the presence/absence of binge eating.

**Results:**

A confirmatory factor analysis revealed that the one-factorial model fits the data best. The results of a linear regression, taking the binge eating score as the dependent variable, showed that higher body dissatisfaction, more alcohol use disorder, higher depression, vomiting to lose weight and starving to lose weight were significantly associated with more binge eating. Higher self-esteem was significantly associated with less binge eating.

**Conclusion:**

The Arabic Version of the BES scale seems to be a reliable tool to be used in Lebanese adolescents for the assessment of binge eating. More body dissatisfaction, lower self-esteem, increased depressive symptoms were associated with more binge eating. We hope this tool will be a reliable one to be used in epidemiological studies and research about eating behaviors/disorders.

**Plain English summary:**

The results showed that higher body dissatisfaction, higher depression, vomiting to lose weight and starving to lose weight were significantly associated with more binge eating. Our study also showed that the Binge Eating Scale is an adapted and validated tool to be used among Lebanese adolescents for the assessment of binge eating. We hope that the study results will help clinicians in the screening and management of Binge Eating behaviors among Lebanese adolescents.

## Background

Binge eating (BE), a manifestation of disordered eating, is defined as an uncontrolled consumption of large of quantities of food [1]. This behavior could be observed in binge eating disorder (BED), bulimia nervosa (BN) and anorexia nervosa (AN) (binge/purge subtype) [2, 3]. In the case of BED, the individual will suffer from multiple BE episodes, in association with feelings of distress, guilt and loss of control sensation during the episodes [4]. This eating disorder is characterized by at least one episode of BE per week for at least 3 months. Unlike other eating disorders such as bulimia nervosa, no compensating behaviors to lose weight are identified, such as purging, over exercising, going on a severe diet or taking medications (diuretics or laxatives) [4]. BED is a common eating disorder among young adolescents, with a lifetime prevalence of 1.6%, followed by BN (0.9%) and AN (0.3%), and is prominent in girls [2].

The available literature related to BE in the Middle East in general and Lebanon specifically, is relatively scarce and/or outdated. When examining the Middle East, the risk of having an episode of BE was highest among Egyptian (1990, *N* = 218) [5] and Kuwaiti populations (2013, *N* = 320, over 80% reported BE) [6]. However, those studies are relatively old and include a relatively small sample of young adults [7]. Other studies showed a BE rate of 4.9% among a Jordanian young adult population [7], while another study indicated a moderate reporting of BE varying between 16.9 and 24.9%, with severe BE between 6.4 and 13.2% among young UAE adults [8].

When focusing on Arabic-speaking adolescent populations, reporting of BE; rates varied between 16.9 and 33% in two different samples of Jordanian adolescents [9, 10], while it was 14% among adolescents in Oman [11]. To the best of our knowledge, no study has evaluated BE in Lebanese adolescents to this date.

A study among Lebanese adults showed an association of BED with several already known BED correlates such as body dissatisfaction, anxiety, depression, sexual abuse, family history of BE and low self-esteem level [12].

The presence of BE in adolescents can be mostly accredited to major eating disorders such as BED and Bulimia, both prevalent in adolescents [13]. Body dissatisfaction (BD) is one of the strongest influencing factors of BE [14]. Literature clearly shows that a reduced body dissatisfaction level in an adolescent will lead to better outcomes in eating disorders treatment [15]. Better control of weight and BE episodes were observed in adolescents as well as a better control of self-esteem levels, anxiety, and depression among these individuals [15]. The role of self-esteem is observed in the development of healthy personality characteristics development [16]. Alongside BD, self-esteem levels also predicted outcome of treatment and a higher self-esteem level further reduced eating disorders development and manifestations in adolescents [17]. Other BE correlates found in the literature include smoking and problematic alcohol consumption. Although waterpipe smoking is prominent in Lebanese adolescents, an approach to underline its correlation to eating disorder and BE specifically is lacking [18].

### Measuring Binge Eating (BE) behavior

The gold standard to assess BE behavior is by interview, using tools such as Eating Disorder Examination. However, it is time consuming and requires a trained clinician, raising the need for a more efficient form of assessment [3]. The development of self-administered, straightforward questionnaires allow a good assessment of individual’s eating disorder symptomatology [3]. Indeed, some behaviors could be easier to assess by the individual himself rather than a secondary party [19]. Moreover, it offers for adolescents the anonymity of their answers, possibly generating more truthful responses among this type of population, in fears of judgment by peers or adults [3]. The nature of BE and the associated loss of control feeling, could be better assessed by validated self-report scales such as the BES [3]. The Binge Eating Scale (BES) covers multiple aspects of eating habits including but not limited to feelings, cognitions and behaviors [20]. It also assesses the impact of environment factors on weight control (such as media pressure, social isolation, and weighing oneself daily) [20].

In a recent systematic review about self-report measures used in the assessment of BE symptoms, the BES showed good internal consistency, an adequate agreement in terms of reproducibility, and very clear floor and ceiling effect, as well as being easy to interpret and differentiate multiple relevant groups within the scale. [3] It was first created for the purpose of detecting BE behaviors in overweight and obese people [20]; it has been later on successfully validated among various ethnic and cultural groups across the world [21–24]. In obese and bariatric candidates, this scale has shown to have great specificity and sensitivity in distinguishing binge from non-binge eaters, in comparison to results obtained from reliable, semi-structured interviews [3]. Additionally, this study has shown high value in non-clinical settings and is used as a treatment outcome assessment tool in some populations [24]. It is worth noting that this scale has been previously validated within the Lebanese adult population and has been found to have good psychometric properties [25]; the results indicated a two-factor model that has a good internal consistency (Cronbach’s alpha = 0.86) [25].

While BE is seen in various disorders, it is rather one of many important manifestations of eating disorders, making its measurement a very important clinical assessment. Thus, the objectives of this study were to (1) validate the BES for use in Lebanese adolescents, and (2) assess the correlates of BE behavior, most importantly self-esteem, body dissatisfaction, anxiety, cigarette and waterpipe dependence, alcohol use disorder, and depression. In terms of correlates, we would assume that higher body dissatisfaction, anxiety, depression, nicotine dependence and problematic alcohol consumption and lower self-esteem would be associated with more BE.

## Methods

### Study design and participants

This cross-sectional study involved a sample of Lebanese adolescents with age ranging from 15 to 18 years old currently residing in Lebanon’s governorates (Beirut, North, South, Bekaa, Mount Lebanon). Excluded from this sample were participants outside the age interval (< 15 years and > 18 years) as well students of Lebanese origin living outside the Lebanese territory. The same methodology is described in previous papers from the same project [26, 27].

### Procedure

The coronavirus pandemic challenged the proper execution of the study due to nationwide lockdown, curfews, required social distancing, and schools’ closing. In response to this new situation, a transition of the study data collection towards an online available questionnaire, was deemed necessary. A “Google Forms” link was therefore, created (https://forms.gle/7J8vMyAyanUugJpf8). Prior to proceeding with the data collection, a pilot study was conducted on 20 students, to assess the duration and technical feasibility as well as fixing misconceptions or misunderstandings related to the questions. The data collection was carried out between May and June 2020 using a snowball technique, delivering a message containing a brief description of our study and the available study link. Later, initial participants were asked to recruit other participants they know from the same age and from all Lebanese governorates.

### Minimal sample size calculation

According to the G-power software and based on an effect size f^2 ^= 2%, an alpha error of 5%, a power of 80%, and taking into consideration 20 factors to be entered in the multivariable analysis, the results showed that a minimum number of 395 was needed to have enough statistical power.

### Forward and back translation

The forward and backward translation method was used on the Rosenberg Self Esteem Scale (RSES) and Eating Disorders Inventory-2 Body Dissatisfaction Subscale (EDI-2-BD) scales in the questionnaire, following international guidelines [28, 29]. The forward translation was done by a clinical psychologist. A psychiatrist performed the back translation. The back-translated English questionnaire was subsequently compared to the original English one, by a committee composed by two psychologists, one psychiatrist and one pharmacist, aiming to discern discrepancies and solve any inconsistencies between the two versions. Revisions of problematic questions were communicated with both translators involved for version updating. The process of forward-back translation was repeated until all ambiguities disappeared.

### Measures

The Arabic questionnaire contained an introductory page explaining the purpose of this study, a confirmation of the anonymity of the participants and a statement about the non-traceability of the responses whatsoever. Furthermore, a consent form, requiring the approval of the parents, was required before proceeding to the questions. The student had the choice to accept or refuse participation, with no financial compensation given.

The survey covered in its first part the sociodemographic factors such as age, gender, governorate, family monthly income, and the house crowding index (HCI), which reflects the socioeconomic status; the HCI is calculated by dividing the number of persons living in the house by the number of rooms excluding the kitchen and bathrooms. A higher HCI score reflects a lower socioeconomic status [30].

In the next section, it covered questions regarding eating habits of the participants that were identified from previous articles [31, 32]: “Do you follow a diet to lose weight?”, “Do you exercise to lose weight?”, “Do you take medication to lose weight?”, “Do you vomit to lose weight?”, “Do you starve yourself to lose weight?”, “Do you weight yourself daily?”, “Do you experience social isolation”, “Do you have a family history of eating disorders?”, “Do you feel pressured by the media to lose weight?” Those questions had a yes/no type of answer.

The final section of the questionnaire contained the internationally validated scales:

#### Binge Eating Scale (BES)

Validated in Lebanon [25], this scale is composed of 16 items, computing a total score from 0 to 46 [20]. People scoring < 17 were classified as having no to mild BE, whereas those who had a score between 18 and 26 and 27 and above were classified as having moderate and severe BE respectively.

#### Rosenberg Self Esteem Scale (RSES)

It is a self-reported, 10-item tool with the target of assessing one’s global self-worth and self-esteem [33]. Items answers vary from strongly disagree to strongly agree. Higher scores indicate higher self-esteem.

#### Eating Disorders Inventory-2 Body Dissatisfaction Subscale (EDI-2-BD)

This subscale assesses the overall body image and specific body parts satisfaction [34]. It contains 9 items questions; the higher the score, the more severe the body dissatisfaction.

#### Patient Health Questionnaire (PHQ-9)

Validated in Lebanon [35], the PHQ-9 [36] is a 9-item instrument developed for screening for the presence of depressive symptoms. Each item is scored on a four-point Likert scale, ranging from 0 (not at all, several days, more than half the days) to 3 (nearly every day). Higher scores reflect more depressive symptoms.

#### Hamilton Anxiety Rating Scale (HAM-A)

The HAM-A scale is 14-item questionnaire, with the target of assessing both psychiatric based anxiety and somatic based anxiety [37]. Higher scores indicate more severe anxiety. This scale has been previously validated among Lebanese adults [31] and among the adolescent population [38].

#### Alcohol Disorder Identification Scale (AUDIT)

This scale, validated in Lebanese adolescents [39], was constructed by the World Health Organization (WHO) [40] with the purpose of profiling alcohol consumption, drinking patterns and drinking related problems. Due to the nature of this study, a self-reported version of this scale was administered, with a score of 8 or more pointing towards the presence of alcohol use disorder.

#### Lebanese Waterpipe Identification Scale (LWDS-11)

The LWDS-11 is composed of 11 items, scored on a four-point Likert scale [41, 42]. Higher scores reflect more waterpipe dependence.

#### Fagerstrom Nicotine Dependence Scale (FTND)

The Fagerstrom nicotine dependence scale [43], validated in Lebanon [44], is a valuable instrument containing 6 items in relation with physical dependence to nicotine assessment. Higher scores reflect higher nicotine dependence.

### Statistical analyses

To assess the internal structure of the BES, we used the confirmatory factor analysis (CFA), which was carried out in Mplus v. 7.2. To evaluate the model fit, we relied on standard recommendations, that is the root mean square error of approximation (RMSEA) statistic and the comparative fit index (CFI) [45]. RMSEA values ≤ 0.06 or CFI values > 0.90 indicate a good-fitting model [45]. The CFA was based on polychoric correlation matrix with Weighted Least Squares with Means and Variances Adjusted estimator (i.e., Mplus WLSMV). We tested two models, present within the literature, that is one-factorial model where a single latent factor is loaded by all items, and a two-factor model, representing behavioral and emotional/cognitive aspects of binge eating [24].

Student *t* and ANOVA tests were used to compare two and three or more means respectively. Pearson correlation test was used to correlate two continuous variables. A linear regression was conducted using the enter method, taking the binge eating score as the dependent variable and all variables that showed a *p* < 0.20 in the bivariate analysis, as independent variables. *P* < 0.05 was considered significant.

## Results

The Cronbach’s alpha values for the scales used in this study were as follows: BES (0.835), RSES (0.768), EDI-2-BD (0.82), PHQ-9 (0.837), HAM-A (0.893), AUDIT (0.888), LWDS (0.622), and FTND (0.632).

The mean age of the participants was 16.67 ± 1.00 years, with 75.7% females. The mean household crowding index was 0.95 ± 0.50. More details about the students can be found in Table 1. The mean BES score was 5.07 ± 4.96 (median = 4; minimum = 0; maximum = 27). The results also showed that 537 (96.8%) adolescents had no or mild binge eating (scores of 17 or less), 17 (3.1%) had possible binge eating (scores between 18 and 26) and 1 (0.2%) adolescent scored 27 on the BES scale and was classified as having binge eating.

**Table 1:** Sociodemographic and other characteristics of the participants (*N* = 555)

Variable	*N* (%)
*Gender*	
Male	135 (24.3%)
Female	420 (75.7%)
*District*	
Beirut	70 (12.6%)
Mount Lebanon	355 (64.0%)
North	84 (15.1%)
South	16 (2.9%)
Bekaa	30 (5.4%)

	**Mean ± SD**
Age (in years)	16.67 ± 1.00
Body Mass Index (kg/m^2^)	22.31 ± 4.07
Household crowding index	0.95 ± 0.50
Cigarette smokers (yes)	19 (3.4%)
Waterpipe smokers (yes)	47 (8.5%)
Alcohol drinking (yes)	549 (98.9%)

### Validation of the BES: confirmatory factor analysis

Results of the CFA revealed that both tested models, that is, one-factorial (χ^2^_(104)_ = 213.23; *p* < 0.001; CFI = 0.964; RMSEA = 0.041[0.033, 0.049]) and two-factorial (χ^2^_(102)_ = 209.16; *p* < 0.001; CFI = 0.965; RMSEA = 0.041[0.033, 0.049]) fitted the data well. The difference in fit indices between these models was however negligible. The χ^2^ test for nested model claimed this difference to be non-significant (χ^2^_(2)_ = 4.07; *p* < 0.131). The behavioral and emotional/cognitive latent factors were furthermore correlated (ρ = 0.95; *p* < 0.001) to the extent to which it was almost impossible to differentiate between them. Thus, the one-factorial structure of the BES in adolescent sample seems to be preferable. The standardized factor loadings for this model are given in the Table 2.

**Table 2:** Standardized factor loadings of the one-factorial measurement model

Item	Factor loading
1. Manner of eating food	.54
2. Degree of loss of control while binge eating	.36
3. Degree of loss of control while eating	.80
4. Food trigger: boredom	.53
5. Impulsive eating	.66
6. Guilt feeling after eating	.67
7. Dieting/Feasting balance	.79
8. Quantity of food digested in one setting	.51
9. Calories balance and control	.60
10. Eating episodes control and balance	.72
11. Stopping an episode of binge eating	.77
12. Social alterations of eating	.72
13. Numbers and planning of meals	.57
14. Obsession and guilt over binge eating	.71
15. Control of food and eating over thoughts and behaviors	.74
16. Recognition of hunger	.59

### Correlates of BES

#### Bivariate analysis

Higher body dissatisfaction, alcohol use disorder, anxiety and depression were significantly associated with more binge eating, whereas higher self-esteem was significantly associated with less binge eating (Table 3). Females compared to males, those who vomit or take medications or starve themselves to lose weight, those who weigh themselves daily and those who feel pressured by media to lose weight had significantly more binge eating (Table 4).

**Table 3:** Bivariate analysis of continuous variables associated with the binge eating score

Variable	Correlation coefficients
Body dissatisfaction	0.35^a^
Self-esteem	− 0.36^a^
Cigarette dependence	0.22
Waterpipe dependence	0.07
Alcohol use disorder (AUDIT score)	0.29^a^
Anxiety	0.26^a^
Depression	0.33^a^
Age	− 0.06
House crowding index	− 0.01
Body Mass Index	0.10

^a^*p* < 0.001; numbers displayed in the table represent correlation coefficients obtained from the Pearson correlation test. Numbers without a sign next to them represent non-significant associations

**Table 4:** Bivariate analysis of categorical variables associated with the binge eating score

Variable	Mean binge eating score	*p*
*Gender*		** < 0.001**
Male	3.78 ± 4.07	
Female	5.68 ± 5.40	
*Following a diet to lose weight*		0.262
No (*N* = 377)	3.99 ± 4.13	
Yes (*N* = 237)	5.88 ± 5.69	
*Exercising to lose weight*		0.262
No (*N* = 197)	4.08 ± 4.72	
Yes (*N* = 417)	5.07 ± 4.89	
*Vomiting to lose weight*		**0.003**
No (*N* = 535)	4.15 ± 4.31	
Yes (*N* = 79)	7.67 ± 6.39	
*Taking medications to lose weight*		**0.008**
No (*N* = 560)	4.31 ± 4.52	
Yes (*N* = 54)	7.38 ± 6.17	
*Starving to lose weight*		**0.009**
No (*N* = 462)	3.94 ± 4.05	
Yes (*N* = 152)	6.93 ± 6.18	
*Weighing yourself daily*		**0.001**
No (*N* = 458)	4.15 ± 4.56	
Yes (*N* = 156)	6.32 ± 5.37	
*Experienced social isolation*		0.115
No (*N* = 419)	4.34 ± 4.68	
Yes (*N* = 195)	5.46 ± 5.13	
*Family history of eating disorders*		0.220
No (*N* = 419)	4.38 ± 4.60	
Yes (*N* = 195)	5.49 ± 5.37	
*Pressured by media to lose weight*		** < 0.001**
No (*N* = 447)	3.68 ± 3.94	
Yes (*N* = 167)	6.86 ± 5.82	

#### Multivariable analysis

The results of a linear regression, taking the binge eating score as the dependent variable, showed that higher body dissatisfaction (*Beta* = 0.16), higher depression (*Beta* = 0.21), alcohol use disorder (*Beta* = 0.24), vomiting to lose weight (*Beta* = 1.71) and starving to lose weight (*Beta* = 1.48) were significantly associated with more binge eating. Higher self-esteem (Beta = − 0.12) was significantly with less binge eating (Table 5).

**Table 5:** Multivariable analysis: linear regression (using the enter model) taking the binge eating score as the dependent variable

Variable	Beta	β	*p*	95% CI
Body dissatisfaction	0.16	0.21	** < 0.001**	0.10–0.22
Self-esteem	− 0.12	− 0.14	**0.001**	− 0.19 to − 0.05
Depression	0.19	0.21	** < 0.001**	0.10–0.28
Anxiety	0.01	0.02	0.730	− 0.03 to 0.05
Alcohol use disorder (AUDIT score)	0.24	0.21	** < 0.001**	0.16–0.31
Gender (females vs males*)	0.66	0.06	0.112	− 0.16 to 1.48
Vomited to lose weight (yes vs no*)	1.71	0.12	**0.005**	0.51–2.92
Medications to lose weight (yes vs no*)	0.57	0.03	0.423	− 0.83 to 1.97
Weigh yourself daily (yes vs no*)	− 0.08	− 0.01	0.850	− 0.95 to 0.78
Pressured by media to change weight (yes vs no*)	0.32	0.03	0.430	− 0.48 to 1.12
Starve yourself to lose weight (yes vs no*)	1.48	0.13	**0.001**	0.57–2.38
Experienced social isolation (yes vs no*)	− 0.34	− 0.03	0.376	− 1.11 to 0.42

*Reference group; Beta = Unstandardized Beta; β = Standardized Beta; CI = Confidence Interval; numbers in bold indicate significant p-values. Nagelkerke *R*^2^ = 34.9%

## Discussion

The results of this study showed that higher BE was associated with higher levels of BD, anxiety/depression as well lower levels of self-esteem, confirming our hypothesis. Moreover, problematic alcoholism was associated with an increase in BE, in agreement with a French adolescents cross sectional study. However, no significant association was found in our sample between BE and waterpipe/cigarette smoking. This result could be due to the small number of smokers in our sample.

### Validation of the BES

The BES has been repetitively validated across different ethnic and cultural groups with continuous consistent and strong results [21, 23, 24]. To our knowledge, no previous attempt has been made towards validating this scale within an adolescent population, and specifically an Arabic speaking one. The initial psychometric properties of the scale suggest a one-factor solution, similar to the results observed in Spanish [24] and French [21] populations, but different from the results obtained in Malay [46] and Lebanese [25] adults (two-factor solution). The internal consistency of the scale in this study (Cronbach’s alpha = 0.835) was similar to the one obtained in the original scale [20] and previous validations in various populations (0.85 in Iran [22], 0.862 in Lebanon [25] and 0.93 in France [21]). Although both factor and confirmatory analysis are satisfying, more psychometric properties of the BES scale (convergent validity with other scales to assess eating disorders (e.g. Eating Attitude Test, test–retest reliability) are still lacking. The survey did not ask about self-reported binge eating (yes/no type of answer). Future studies should focus on assessing more psychometric properties of the BES by collecting data about more closely related measures to the scale and also examine the scale’s reliability within this population in terms of test–retest. Consequently, the Arabic version of the Binge Eating Scale (BES) seems to be a reliable tool for screening for the presence of binge eating among Lebanese adolescents.

Concerning the rate of binge eating, the results showed that 17 (2.8%) students had possible binge eating and 1 (0.2%) adolescent had binge eating. A study with a similar sample size and average age of participants reported a prevalence of 1% of BE among participants [47]. On the other hand, a study assessing BE among 2000 adolescents in Mumbai reported a high prevalence of binge eating behavior (50%), with 36% reporting severe binge eating [48]. However, these estimates need to be interpreted with caution. We reported 1% of BE according to our sample and 50% of BE according to its scores in the Mumbai study population [48]; this might be due to the convenient sample collected in the study (because of the pandemic and restrictions of face-to-face meetings) as well as gender [49] and cultural [50] differences regarding attitudes towards eating. Future studies should resolve this problem by recruiting a random sample of adolescents.

### Correlates of binge eating

Higher depression and body dissatisfaction were significantly associated with more BE, in line with previous studies in adolescents [51–53]; this association with BE could be partially explained by the severity of BD in the individual, which was found to severely affect and predict depression [54]. In addition, this association could be the result of a reciprocal relationship between depression and BE with one factor being the cause or result of the other [55, 56], while some studies suggested the possibility of them sharing common risk factors [57, 58]. For example, feeling worthless could be a shared basis for the development and maintenance of depression, and the root for the perceived sense of shame with regard to body self-evaluation [55].

Higher BD was associated with more BE, consistent with various other studies in different populations [2, 25]. In a pathway model established by Tuschen Cafier and Hilbert [59], external and internal stressors triggering BE were highlighted (low self-esteem, exposure to food, impulsive behavior, tensions). Moreover, weight management and body image dissatisfaction were considered as major risk factors for the development, evolution and treatment of binge eating [59]. Body dissatisfaction is a lifelong predictor of eating disorders [27, 60, 61] and specifically binge eating [62, 63].

Our results indicated that lower self-esteem was associated with more binge eating behaviors among adolescents, in line with other studies [64–67]. Self-esteem could be either a cause to or a consequence of body dissatisfaction and body image disturbance [54, 68]. Low self-esteem was found to be a source of negative emotions and negative image towards the self, in addition to increasing the risk of eating disorders [69]. Indeed, global self-esteem is representative of inner evaluation towards oneself and reflects negative affect, with eating disorder patients having a level of self-esteem regulation based on their perceived unsatisfactory weight and shape acceptance [70].

Vomiting to lose weight was associated with more BE behaviors, a condition found in certain eating disorders such as BN and subtypes of AN but not binge eating disorder [4]. The presence of vomiting to lose weight has long been described in BE pathology [71, 72], as part of weight controlling behaviors associated with disordered eating. Studies have shown that in adolescents, vomiting to lose weight is highly prevalent as weight control behaviors [73–75].

Our analysis revealed that starving to lose weight was associated with more binge eating behavior, in line with previous findings that showed that a purposeful decrease in calories/meals intake was found in various eating disorders [76]. Starvation to lose weight is a radical form of weight loss maneuver, part of dietary restraint, and was found to be a maintenance factor of binge eating behaviors in various other studies [65, 77].

### Clinical implications

The results described above, although acknowledged previously in the literature, reconfirm the underlying psychiatric burden of BE in Lebanese adolescents. The establishment of these relationships broaden the profile of a person who engages in binge eating and may offer a better management and treatment of any underlying conditions that could be present. These results will allow us gain a better perspective about BE among Lebanese adolescents, guiding future studies in this field. The results also emphasize on the lower rate of BE in the Lebanese society compared to Western cultures. Binge eating behaviors, if present within the adolescent population in Lebanon, are mainly driven by an elevated body dissatisfaction level, depression, and lower self-esteem. This underlines the need to address eating disorders more frequently during adolescence and advise various society members such as teachers, parents, and health care providers on the issue. Additionally, awareness among adolescents should be initiated since eating disorders pose some serious health adverse effects on the body and mind.

### Limitations and strengths

This research used a cross-sectional study design and therefore cannot underline causalities and relationships between the above studied variables. The scales used for evaluation of some research parameters were not fully validated in the Arabic language and are used for screening purposes of disordered psychiatric behaviors; therefore, they could not confirm the clinical diagnosis and cannot replace the need for a clinical interview. The snowball sampling technique and respondent driven strategies used for achieving the sample size, alongside external factors such as the ongoing COVID19 pandemic and its resulting nationwide lockdown presented a challenge for proper data collection. The use of this technique could lead to a source of information bias mainly in relation of understanding the questions, accurately estimating symptoms, resulting in possible inaccuracy. A disequilibrium in our sample was a numerical superiority in female respondents compared to their male counterparts and in terms of the higher percentage of alcohol drinking and low percentages of cigarette and waterpipe smoking. Furthermore, the results of this study may not be representative of the entire population since respondents had a high school level of education. The COVID-19 pandemic and its consequent lockdowns were associated with higher mental health issues (depression, anxiety, stress, obsession, etc.) among the Lebanese population [78], as well as more disordered eating [79]. The economic crisis, lack of clean water, recurrent power failure, and waste mismanagement and Syrian refugees immigration to Lebanon [80, 81] have also impacted psychological behaviors, possibly affecting our findings. Finally, a residual confounding bias is also possible since not all factors associated with binge eating were taken into consideration in this study. On another hand, this study is unique in the Arabic region (and the first in Lebanon) in addressing binge eating disorders in an Arabic adolescent population, with a large sample size distributed across major districts. It can be useful as preliminary template to further develop future studies.

## Conclusion

This study has acknowledged some correlates of BE among adolescents. Additionally, the validation of the Arabic BES scale is a crucial contribution to the relatively scarce published literature available regarding binge eating behavior and represents a step forward toward characterizing BE’s presence in the region, a topic poorly addressed in the Arabic community. Finally, the study results offer a promising perspective into continuation of research; possibly with an interview-based approach, with the help of clinicians in order to better tailor screening and management of Binge Eating behaviors in Lebanese adolescents.

## Data Availability

All data generated or analyzed during this study are not publicly available to maintain the privacy of the individuals’ identities. The dataset supporting the conclusions is available upon request to the corresponding author.

## Abbreviations

BES: Binge Eating Scale
BE: Binge eating
BED: Binge eating disorder
BN: Bulimia nervosa
AN: Anorexia nervosa
HCI: Household crowding index
RSES: Rosenberg Self Esteem Scale
EDI-2-BD: Eating Disorders Inventory-2 Body Dissatisfaction Subscale
PHQ-9: Patient Health Questionnaire
HAM-A: Hamilton Anxiety Rating Scale
AUDIT: Alcohol Disorder Identification Scale
WHO: World Health Organization
LWDS-11: Lebanese Waterpipe Identification Scale
CFA: Confirmatory factor analysis
CFI: Comparative fit index
WLSMV: Weighted least squares with means and variances adjusted estimator
